# Preliminary Psychometric Evaluation of the Economic Living Standards Index Short Form (ELSI-SF) Among Spanish Older Adults

**DOI:** 10.3390/healthcare14172673

**Published:** 2026-08-22

**Authors:** Celia Álvarez-Bueno, María Eugenia Visier-Alfonso, Mar de Miguel-Brox, Blanca Zuheros-Lara, María Frontelo-García, Beatriz Rodríguez-Martín

**Affiliations:** 1 Age-ABC Group, Health and Social Research Center, Universidad de Castilla-La Mancha, 16002 Cuenca, Spain; celia.alvarezbueno@uclm.es (C.Á.-B.); blanca.zuheros@alu.uclm.es (B.Z.-L.); maria.frontelo@alu.uclm.es (M.F.-G.); beatriz.rmartin@uclm.es (B.R.-M.); 2 Facultad de Ciencias de la Salud, Universidad Autonoma de Chile, Talca 7500912, Chile; 3 Health and Social Research Center, Universidad de Castilla-La Mancha, 16002 Cuenca, Spain; mariaeugenia.visier@uclm.es; 4 Department of Nursing, Physiotherapy and Occupational Therapy, University of Castilla-La Mancha, 45600 Toledo, Spain

**Keywords:** aging population, material well-being, psychometric validation, living standards, older adults

## Abstract

**Highlights:**

**What are the main findings?**

- This study provides preliminary psychometric evidence for the Spanish ELSI-SF in older adults.
- The total score showed acceptable reliability, while two subscales showed limited reliability and the four-factor structure received only partial support.

**What are the implications of the main findings?**

- The total ELSI-SF score may be useful for assessing material living standards in relatively socioeconomically advantaged Spanish older adults.
- Further validation in larger and more socioeconomically diverse samples is needed, particularly among older adults experiencing material deprivation.

**Abstract:**

**Objective:** To examine the reliability, construct validity, and discriminative capacity of the Spanish version of the Economic Living Standards Index Short Form (ELSI_SF_) in a sample of older adults in Spain. **Methods:** This analysis was conducted using baseline data from 133 participants (mean age = 66.2 years, SD = 5.6; 53.8% women) recruited from community health and social centers in the provinces of Cuenca, Albacete, and Toledo within the PEPE cohort. Participants completed the ELSI_SF_ (25 items), sociodemographic measures, the SF-12 Health Survey, and the International Fitness Scale (IFIS). Internal consistency was assessed using Cronbach’s alpha. Convergent validity was examined through correlations between ELSI_SF_ scores and education, occupation, physical and mental health, and perceived fitness. Discriminative ability for health-related variables was analyzed using ANOVA and partial η^2^. The internal structure was explored using exploratory factor analysis (EFA) and subsequently examined against the original four-factor theoretical model using confirmatory factor analysis (CFA), based on polychoric correlations and estimation methods appropriate for ordinal data. **Results:** The mean ELSI_SF_ score was 23.0 ± 3.9, with most participants classified as having comfortable or good living standards. Internal consistency was acceptable for the total scale (α = 0.779), whereas the ownership restrictions (α = 0.441) and self-rating (α = 0.319) subscales showed low reliability. Significant associations were observed between the ELSI_SF_ total score and selected socioeconomic and health-related measures. Factor-analytic findings provided only partial support for the original four-domain structure. Although CFA showed favorable CFI, TLI, and RMSEA values, the elevated SRMR and instability of the self-rating factor warrant cautious interpretation of the factorial solution. **Conclusions:** The Spanish ELSI_SF_ provides preliminary psychometric evidence supporting the use of its total score among relatively socioeconomically advantaged older adults. However, limitations in subscale reliability, factorial stability, and the restricted socioeconomic variability of the sample preclude definitive validation across the full spectrum of material living standards. Further evaluation in larger, independent, and more socioeconomically heterogeneous samples, particularly including older adults experiencing material deprivation, is required.

## 1. Introduction

According to projections from the World Health Organization (WHO), population aging will become one of the most significant demographic phenomena of the twenty-first century. By 2050, one in six people globally is expected to be aged 65 years or older, representing approximately 16% of the world’s population [1]. In Europe, this demographic transformation is accompanied by declining birth rates and improved social and health systems, leading to profound shifts in population structures and increasing social and economic pressures on governments [2,3].

The increased demand for healthcare and social support services is related to the decline in physical and mental function inherent to aging that poses a potential health risk [4]. Moreover, older adults may live under diverse economic conditions and social determinants that tend to exacerbate socioeconomic inequalities and limit opportunities for aging satisfactorily [4,5]. In addition, older adults could face qualitative changes in needs and preferences that have a direct impact on quality and are relevant to communicable and non-communicable diseases. This has intensified interest in how to support older adults in achieving a successful and meaningful aging process.

In this regard, assessing living standards provides an insightful perspective on the economic and social inequities that influence aging trajectories, reflecting opportunities to age positively and to participate actively within communities [5]. The capability approach, proposed by Sen (1999), conceptualizes the freedom to live the life one values as a function of access to resources and life-course advantages that support health and well-being [6]. Higher living standards are associated with better mental and physical health outcomes and lower mortality rates [5,7]. Traditionally, living conditions have been assessed through proxies such as quality of life, income, or educational attainment; however, none of these indicators fully capture the real impact of socioeconomic status on lifestyle and material well-being [6,8]. While quality of life measures focus on intangible aspects of subjective well-being, standard-of-living indices offer a more direct and material perspective [6,8,9,10].

To address these limitations, the Economic Living Standards Index (ELSI) and its Short Form (ELSI_SF_) were developed in New Zealand as brief, multidimensional measures of material well-being [11]. ELSI_SF_ evaluates four domains: ownership restrictions, social participation restrictions, economizing, and self-rating of one’s economic situation. It has demonstrated good internal consistency (α = 0.88) and significant correlations with income, financial stress, and health indicators, confirming its validity and reliability in the reference population [12]. More recent studies have supported its cross-cultural applicability and psychometric robustness in diverse aging populations [13].

Despite these findings, given the absence of previous psychometric evidence for the Spanish version of the ELSI_SF_, the present study should be understood as an initial evaluation of its measurement properties in a community-based sample of Spanish older adults.

## 2. Materials and Methods

### 2.1. Design, Study Participants, and Procedures

This study analyzed data from 133 older adults (53.8% women) aged between 60 and 75 years (M = 66.2, SD = 5.6), who were part of a broader prospective longitudinal project—the PEPE Cohort Study—designed to identify profiles of retirees and the determinants of successful retirement [14]. Participants were recruited from the Spanish provinces of Cuenca, Albacete and Toledo between January and December 2023.

Potential participants were identified through lists provided by local health and social care centers. Eligible individuals were contacted by the research team via telephone and invited to participate in the study. Additional recruitment was conducted through flyers and posters displayed in community spaces where older adults are typically engaged in leisure or social activities.

Inclusion criteria were: (1) being aged 60 years or older, and (2) either approaching retirement within six months or having retired within the previous 12 months. Exclusion criteria included being a housewife or early retiree due to health or family-related issues. Those who agreed to participate received the study questionnaires by post or email.

The study was conducted in accordance with the Declaration of Helsinki, and ethical approval was obtained from the Clinical Research Ethics Committee of the “Virgen de la Luz” Hospital. All participants were informed about the study objectives and procedures and provided written informed consent before data collection.

### 2.2. Measurements

Sociodemographic variables were collected using a self-administered questionnaire. Marital status was classified as single, married, divorced, widowed, or cohabiting partner. Educational level was recorded as the highest level attained and categorized into no literacy, no formal studies, primary education, secondary education, vocational or pre-university studies, university degree, or postgraduate degree (Master’s/PhD). Occupational status was classified following the Spanish Society of Epidemiology scale [15] into unemployed, employed, self-employed or company manager, pre-retiree, or retiree.

Economic living standards were assessed using the Economic Living Standards Index—Short Form (ELIS_SF_) [11], a 25-item instrument designed to measure the economic standard of living across four domains: ownership restrictions, social participation restrictions, economizing, and self-rating of one’s economic situation. Total scores range from 0 to 31, with higher scores indicating better living standards. Standard categories were applied as follows: (1) severe hardship (0–8), (2) significant hardship (9–12), (3) some hardship (13–16), (4) fairly comfortable (17–20), (5) comfortable (21–24), (6) good (25–28), and (7) very good (29–31). The lowest three categories were considered indicative of material hardship.

Health-related quality of life was measured using the 12-Item Short Form Health Survey (SF-12) [16]. This scale evaluates eight subdomains—physical functioning, physical role, bodily pain, general health, vitality, social functioning, emotional role, and mental health—which yield two composite scores: Physical Component Summary (PCS) and Mental Component Summary (MCS). Higher scores indicate better health-related quality of life.

General physical fitness was assessed using the first item of the Spanish validated version of the International Fitness Scale (IFIS) [17], which asks participants to rate their overall physical fitness on a five-point scale ranging from very poor to very good, compared to peers of the same age.

### 2.3. Statistical Analysis

To validate the ELSI_SF_ into the Spanish cultural context, this study adopted the back-translation technique proposed by Brislin [18]. Two bilingual postgraduates in nursing and one anthropologist made the forward translation of the original ELSI_SF_. Then, two postgraduates in nursing who were bilingual in English made the back-translation of the initially-translated Spanish version. Based on the comparison of the back translation and the original ELSI_SF_, the expert panel that consisted of three expert researchers made modifications to the initial Spanish version. Then, the second version of ELSI_SF_ was evaluated by three older adults to assess its comprehension and acceptability. Any discrepancies were resolved to produce the final version of ELSI_SF_.

Descriptive statistics (frequencies and percentages for categorical variables; mean and standard deviation [SD] for continuous variables) were calculated to describe the sample. Only participants with complete data for the variables included in the present analyses were retained. Accordingly, all analyses were conducted using complete cases, and no missing values were imputed.

Reliability was examined using Cronbach’s alpha for the total ELSI_SF_ and for each subscale. For each item, Cronbach’s alpha if item deleted was computed to assess the contribution of individual items to internal consistency.

For the analysis of convergent validity, Spearman’s rank correlation coefficients were calculated between the ELSI_SF_ total score and subscales and sociodemographic and health-related variables (education, occupation, quality of life, and physical fitness).

To evaluate discriminant validity, one-way ANOVA tests were performed to compare ELSI_SF_ scores across categories of physical and mental health. Partial eta squared (η^2^) was reported as an effect size index, with benchmarks of 0.01 (small), 0.06 (medium), and 0.14 (large) [19]. Statistical significance was set at *p* < 0.05.

The internal structure of the ELSI_SF_ was examined using exploratory and confirmatory factor analyses (EFA and CFA), based on polychoric correlations and estimated with methods appropriate for ordinal data, WLSMV (weighted least squares mean and variance-adjusted) [20,21]. The software R and the lavaan package were used.

## 3. Results

### 3.1. Participant Characteristics

As shown in Table S1, complete data for all variables included in the present analyses were available for all 133 participants; therefore, no participants were excluded due to missing data and no imputation procedures were required. Of the participants, 53.8% were women and 73.9% were married or cohabiting. Nearly half (46.2%) had completed primary education, and 44.6% were already retired. Most participants reported a comfortable or good living standard. The mean for total ELSI_SF_ score was 23.0 (SD = 3.9), with the following distribution across categories: no severe hardship (0%), significant hardship (0.8%), some hardship (7.9%), fairly comfortable (14.3%), comfortable (34.9%), good (40.5%), and very good (1.6%).

### 3.2. Reliability

As presented in Table 1, the ELSI_SF_ showed adequate overall reliability (Cronbach’s α = 0.779). Subscale analyses revealed acceptable internal consistency for social participation restrictions (α = 0.685) and economizing (α = 0.744), whereas the ownership restrictions (α = 0.441) and self-rating (α = 0.319) subscales demonstrated low reliability. The analysis of alpha if item deleted indicated that removing item 24 slightly improved the total scale’s reliability (α = 0.789) (Table S2).

### 3.3. Convergent Validity

As reported in Table S3, the total ELSI_SF_ score was positively and significantly correlated with educational attainment, occupational status, physical health, mental health, and self-reported physical fitness (all *p* < 0.05). However, occupation was not associated with ELSI_SF_, but it might be due to the homogeneity of the study sample.

### 3.4. Discriminant Validity

Table S4 presents the results of the mean comparisons of ELSI_SF_ scores according to physical and mental health categories. Participants with higher ELSI_SF_ scores reported significantly better physical and mental health, with a medium effect size for mental health (η^2^ = 0.07). The items showing the greatest discriminative capacity were item 14 (physical health; η^2^ = 0.06) and item 12 (mental health; η^2^ = 0.07). These findings provide preliminary evidence that ELSISF scores differ across selected categories of perceived health and well-being in this sample.

### 3.5. Exploratory Factor Analysis (EFA)

An EFA was conducted to examine the internal structure of the ELSI_SF_. Given the ordinal nature of the items and the presence of ceiling effects, analyses were based on a polychoric correlation matrix, and factors were extracted using the minimum residual (MINRES) method with oblique (oblimin) rotation. Parallel analysis suggested a seven-factor solution. However, this result was interpreted with caution because several items showed highly skewed distributions and very limited variance, which may affect polychoric correlations and factor-retention decisions. The four-factor solution was therefore examined because it corresponded to the theoretical structure proposed by Jensen et al. (2005) [11] while recognizing that alternative factorial structures cannot be definitively ruled out in the present sample. The four-factor solution (Table 2) accounted for 50.7% of the total variance. The first three factors explained 17.1%, 14.6%, and 12.4% of the variance, respectively, whereas the fourth factor accounted for a smaller proportion (6.6%; Table 3).

Overall, the pattern of factor loadings was partially consistent with the theoretical domains of the ELSI_SF_. Items related to ownership restrictions and social participation restrictions showed generally high loadings on their respective factors, whereas the economizing and self-rating domains displayed a weaker and less clearly defined structure. Several items exhibited cross-loadings or failed to reach the minimum loading threshold (≥0.30), and the fourth factor was defined by relatively few items. Inter-factor correlations were moderate among the first three factors (r ≈ 0.35–0.43), while the fourth factor showed low correlations with the remaining factors. These findings suggest that although a four-factor structure is plausible, some dimensions—particularly those represented by fewer items or limited variability—are less stable in this sample.

### 3.6. Confirmatory Factor Analysis (CFA)

A CFA was subsequently conducted to test the original four-factor model of the ELSI_SF_, comprising ownership restrictions, social participation restrictions, economizing, and self-rated material living standards. The CFA was estimated using a robust WLSMV, with all items specified as ordered categorical variables.

The model demonstrated acceptable fit according to several global fit indices (CFI = 0.98; TLI = 0.98; RMSEA = 0.046, 90% CI [0.029, 0.060]). Standardized factor loadings were statistically significant (*p* < 0.05) and generally high for the ownership, social participation, and economizing domains. In contrast, the self-rating domain showed heterogeneous loadings, with two items displaying low standardized loadings (<0.30) and one item exhibiting a very high loading, indicating that this factor was largely driven by a single indicator (see Table 4).

Despite the favorable values of CFI, TLI, and RMSEA, the standardized root mean square residual (SRMR = 0.276) indicated poor reproduction of the observed correlation matrix (Table 5). In addition, estimation warnings suggested issues of model identification and near-perfect polychoric correlations between some dichotomous items, particularly within the social participation domain. Inspection of item distributions revealed extreme ceiling effects, with several dichotomous items showing minimal variability, a condition that can lead to unstable polychoric correlations and inflated factor covariances. Overall, the CFA provides partial support for the theoretically proposed four-factor structure, although the stability of some dimensions, particularly the self-rating factor, appears limited within the present sample.

**Figure 1:**
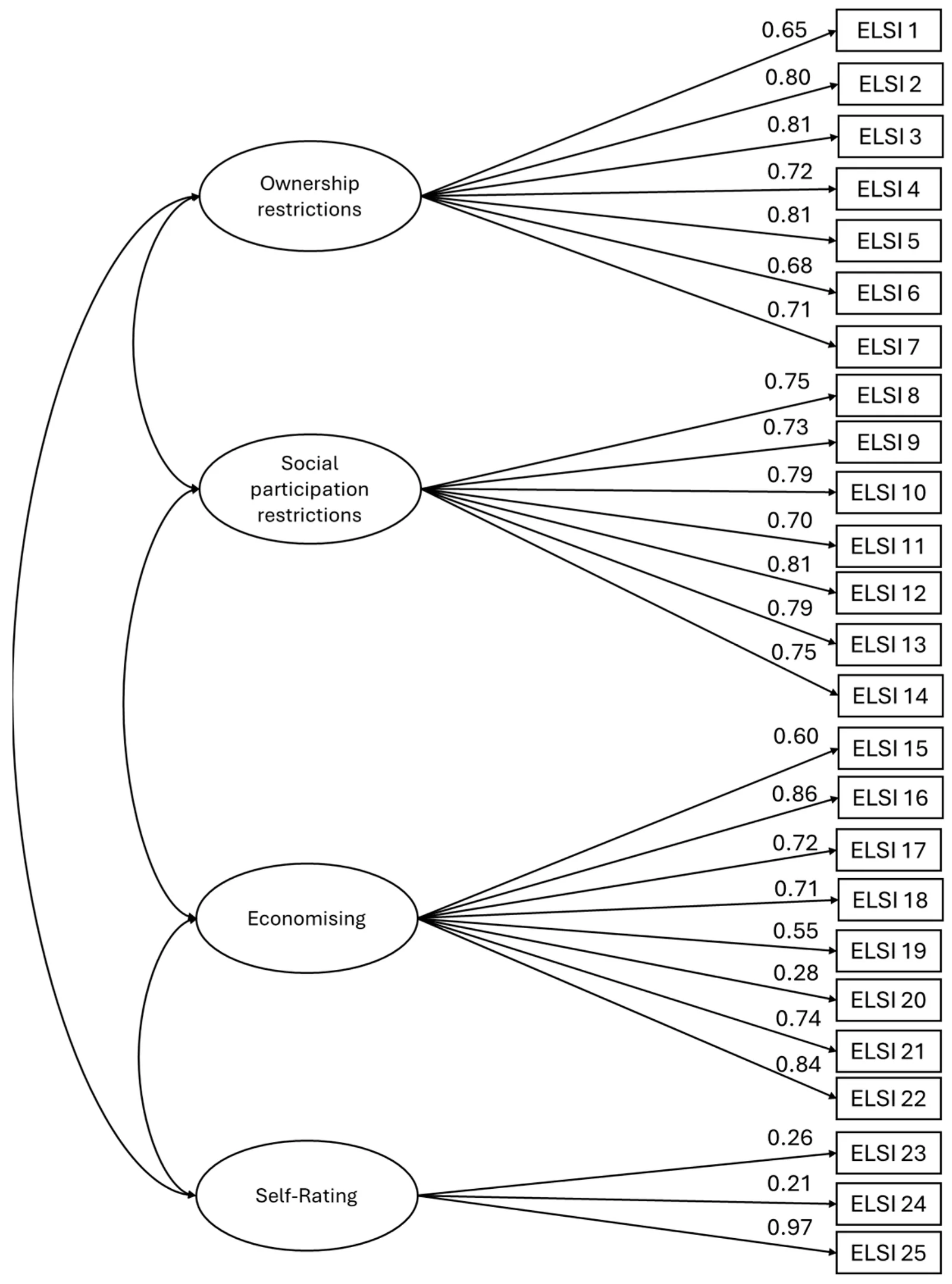
Confirmatory factor analysis model of the ELSI_SF_. Note. Standardized parameter estimates from the four-factor CFA model of the Economic Living Standards Index Short Form (ELSI_SF_). The model was estimated using the WLSMV estimator with ordered categorical indicators. Latent factors were allowed to correlate.

Items related to ownership restrictions and social participation restrictions generally showed moderate to high loadings on distinct factors, whereas the economizing and self-rating domains exhibited a less clearly defined structure. Several items demonstrated cross-loadings or failed to reach the conventional loading threshold (≥0.30), and the fourth factor was defined by a relatively small number of items. Inter-factor correlations were moderate among the first three factors (r ≈ 0.35–0.43), while correlations involving the fourth factor were notably weaker (Figure 1).

## 4. Discussion

This study provides preliminary evidence supporting the reliability and validity of the Spanish version of the ELSI_SF_ among relatively socioeconomically advantaged older adults. Although the total ELSI_SF_ demonstrated acceptable internal consistency, the ownership restrictions and self-rating subscales showed limited reliability. Therefore, the total score appears to represent the most robust summary measure in the present sample, whereas the interpretation of individual subscales should be undertaken cautiously until further psychometric evidence becomes available.

While the overall reliability of the scale was satisfactory and within the range considered adequate for validation studies, internal consistency varied across subscales. In particular, the social participation restrictions and economizing subscales showed acceptable reliability, suggesting that these domains consistently capture relevant aspects of material well-being and economic management strategies. In contrast, lower reliability was observed for other subscales, warranting cautious interpretation of subscale-level findings. Thus, the present results primarily support the reliability of the total ELSI_SF_ score rather than the reliability of each of its individual dimensions.

Although the present findings support the psychometric performance of the Spanish ELSI_SF_, they should be interpreted as preliminary evidence rather than definitive validation. The socioeconomic profile of the study population, characterized by predominantly comfortable living standards and very limited representation of individuals experiencing material hardship, restricted the variability of responses and limited evaluation of the instrument across the full continuum of material deprivation. Consequently, further studies including larger and more socioeconomically diverse samples are required before generalizing these findings to the broader Spanish older population. In particular, the discriminatory performance of the instrument among older adults experiencing substantial material deprivation remains to be established.

Regarding structural validity, the factor-analytic findings provide only partial support for the original four-domain structure of the ELSI_SF_. Although several global CFA fit indices (CFI, TLI and RMSEA) suggested acceptable model fit, the markedly elevated SRMR, together with estimation warnings, near-perfect polychoric correlations, and the instability observed in the self-rating factor, indicates that the confirmatory solution should be interpreted cautiously. In particular, two self-rating items showed standardized loadings below 0.30, whereas the remaining item displayed a very high loading, suggesting that this latent dimension was not robustly represented in the present sample. These findings therefore do not provide definitive confirmation of the original four-factor model.

Furthermore, parallel analysis initially suggested a seven-factor solution. This result was interpreted cautiously, because several items showed extreme ceiling effects and sparse response distributions, conditions that may affect polychoric correlations and the number of factors suggested for retention. The four-factor solution was therefore examined because it corresponded to the theoretical structure of the original ELSI_SF_; however, the present data do not allow alternative factorial structures to be definitively ruled out. Future studies using larger and more socioeconomically heterogeneous samples should compare alternative factor solutions and independently evaluate the stability of the original four-domain model.

Overall, these findings suggest that the total ELSI_SF_ score may currently provide a more robust representation of material living standards than some of its individual subscale scores in this population. Assessing living standards from both economic and social perspectives is essential for designing interventions that promote active, autonomous, and equitable aging. In this context, the ELSI_SF_ may provide useful information for identifying material and social needs and examining how these factors relate to well-being in later life; however, its application for individual subscale interpretation or across the full socioeconomic spectrum requires further psychometric evaluation.

One aspect deserving particular attention is the low reliability observed in the self-rating subscale. These results may be explained by several methodological and conceptual factors. Firstly, this subscale comprises a small number of items, which naturally tends to lower alpha coefficients. Secondly, the items address subjective perceptions of satisfaction and living standards, which can be influenced by individual factors, cultural expectations, or social desirability bias. Moreover, in a sample with predominantly comfortable living conditions, response variability is likely to be limited, thereby reducing covariance between items. Nevertheless, the inclusion of this subscale remains relevant, as it provides complementary information regarding the individual’s personal assessment of their economic situation—an aspect not fully captured by the more objective dimensions of the instrument. For future adaptations, it would be advisable to review the item formulation or include additional measures that more stably assess subjective perceptions of material well-being.

With regard to the sociodemographic characteristics of the sample, the majority of participants were married or cohabiting, had completed primary education, and were either pre-retired or retired. The distribution across the ELSI-SF categories indicated that more than 75% of the sample fell within the “comfortable”, “good”, or “very good” living standard levels, and none reported “severe hardship”. Only 8.7% experienced some form of economic difficulty. This pattern suggests that the sample had a relatively stable living standard and adequate access to material and social resources. However, such homogeneity may have limited the study’s capacity to capture variability at the lower end of the economic continuum, potentially constraining the generalizability of the findings to more vulnerable or lower-income populations. Thus, these results should be interpreted with caution as preliminary evidence. Moreover, because recruitment was conducted primarily through health, social, and community settings, selection mechanisms may have contributed to the relatively advantaged socioeconomic profile observed in the study population.

The evidence regarding convergent and discriminant validity should also be considered preliminary. Although associations with educational attainment, occupational status, health-related quality of life, and perceived physical fitness provide some support for construct validity, the range of external criteria available within the PEPE cohort was limited. In particular, objective or complementary indicators such as household income, wealth, financial strain, or material deprivation were not available. Future studies should therefore examine the relationship between ELSI_SF_ scores and a broader set of socioeconomic indicators and external measures of material well-being.

This study presents several limitations that should be considered when interpreting the results. Firstly, its cross-sectional design prevents causal inferences between perceived living standards and health or well-being outcomes. Secondly, self-reported data may be subject to recall or social desirability bias, particularly regarding economic satisfaction and quality-of-life measures. Third, the relatively small sample size (n = 133) represents a limitation, particularly for subscale-level analyses. Although robust estimation procedures suitable for ordinal data were employed, the relatively small sample limits the stability and generalizability of the factor-analytic results. Furthermore, both EFA and CFA were conducted using the same sample. Consequently, the CFA cannot be regarded as an independent confirmation of the exploratory structure, and the resulting factorial solution may partly reflect sample-specific characteristics. Independent cross-validation in a larger sample is therefore needed.

In addition, the sample showed pronounced ceiling effects, with over 75% of participants classified in the “comfortable”, “good”, or “very good” categories and no cases of severe hardship. This restricted range substantially reduced score variability, likely attenuating reliability estimates and limiting the stability of factor-analytic and correlational results. Accordingly, findings—especially at the subscale level—should be interpreted as preliminary and sample-specific. Additionally, the relatively small sample size limited the feasibility of conducting population-level subgroup analyses by employment status and educational level, as further stratification would have resulted in reduced statistical power and unstable estimates; future studies with larger samples should address these comparisons. The psychometric evaluation was also limited to internal consistency, structural validity, and associations with selected external variables. Other relevant measurement properties, including test–retest reliability, measurement invariance, responsiveness, and additional reliability indices, should be examined in subsequent studies. Finally, the low internal consistency observed in the ownership restrictions and self-rating subscales should be interpreted with caution, as these subscales were evaluated in a relatively small and socioeconomically homogeneous sample. Future studies should incorporate larger and more heterogeneous samples of older adults from diverse socioeconomic and regional contexts to further examine the instrument’s sensitivity in settings characterized by greater material deprivation or inequality.

## 5. Conclusions

Overall, the present findings provide preliminary support for the use of the total Spanish ELSI_SF_ score among relatively socioeconomically advantaged older adults. However, limitations in subscale reliability, factorial stability, and socioeconomic representativeness indicate that further validation in larger, independent, and more heterogeneous samples is required before its measurement properties can be considered firmly established.

## Figures and Tables

**Table 1:** Cronbach’s alpha of the ELSI_SF_ questionnaire and subscales.

Variable	Cronbach’s Alpha	95% CI
1. ELSI_SF_ Total	0.779	0.721; 0.830
2. ELSI_SF_ Ownership restrictions	0.441	0.282; 0.575
3. ELSI_SF_ Social participation restrictions	0.685	0.561; 0.740
4. ELSI_SF_ Economizing	0.744	0.672; 0.805
5. ELSI_SF_ Self-ratings	0.319	0.091; 0.498

CI: Confidence interval.

**Table 2:** Exploratory factor analysis of the ELSI_SF_ (polychoric correlations, MINRES, oblimin rotation).

Item	Factor 1	Factor 2	Factor 3	Factor 4
ELSI_SF_ 1	0.781			
ELSI_SF_ 2	0.581	−0.494		0.330
ELSI_SF_ 3	0.815			
ELSI_SF_ 4	0.635			
ELSI_SF_ 5	0.735			
ELSI_SF_ 6	0.801			
ELSI_SF_ 7		0.663		0.431
ELSI_SF_ 8		0.784		
ELSI_SF_ 9		0.852		
ELSI_SF_ 10	0.347	0.905		
ELSI_SF_ 11			0.899	
ELSI_SF_ 12			0.543	
ELSI_SF_ 13	0.332		0.367	0.558
ELSI_SF_ 14			0.696	
ELSI_SF_ 15	0.317		0.793	
ELSI_SF_ 16		0.451		
ELSI_SF_ 17			(<0.30)	
ELSI_SF_ 18	0.333	0.445		
ELSI_SF_ 19	0.485	0.355		
ELSI_SF_ 20			0.491	
ELSI_SF_ 21	0.337		0.310	
ELSI_SF_ 22			0.380	
ELSI_SF_ 23			(<0.30)	
ELSI_SF_ 24			0.335	
ELSI_SF_ 25	0.435			

Note. Only factor loadings ≥ 0.30 are shown. Extraction method: minimum residuals (MINRES). Rotation: oblimin. Factors were allowed to correlate.

**Table 3:** Variance explained and factor correlations in the EFA.

Factor	SS Loadings	Proportion of Variance	Cumulative Variance
Factor 1	4.264	0.171	0.171
Factor 2	3.658	0.146	0.317
Factor 3	3.100	0.124	0.441
Factor 4	1.649	0.066	0.507

**Table 4:** Standardized factor loadings from the CFA of the ELSI_SF_.

Latent Factor	Item	Standardized Loading
Ownership restrictions	ELSI_SF_ 1	0.647
	ELSI_SF_ 2	0.796
	ELSI_SF_ 3	0.806
	ELSI_SF_ 4	0.716
	ELSI_SF_ 5	0.809
	ELSI_SF_ 6	0.683
	ELSI_SF_ 7	0.714
Social participation restrictions	ELSI_SF_ 8	0.752
	ELSI_SF_ 9	0.732
	ELSI_SF_ 10	0.786
	ELSI_SF_ 11	0.697
	ELSI_SF_ 12	0.807
	ELSI_SF_ 13	0.786
	ELSI_SF_ 14	0.751
Economizing	ELSI_SF_ 15	0.600
	ELSI_SF_ 16	0.860
	ELSI_SF_ 17	0.721
	ELSI_SF_ 18	0.715
	ELSI_SF_ 19	0.546
	ELSI_SF_ 20	0.281
	ELSI_SF_ 21	0.744
	ELSI_SF_ 22	0.844
Self-rating	ELSI_SF_ 23	0.263
	ELSI_SF_ 24	0.219
	ELSI_SF_ 25	0.973

Note. Standardized factor loadings are reported. All loadings were statistically significant (*p* < 0.05). The CFA was estimated using the WLSMV estimator with all items specified as ordered categorical variables. Items with standardized loadings below 0.30 are shown for completeness.

**Table 5:** Model fit indices for the CFA of the ELSI_SF_.

Index	Value
CFI	0.984
TLI	0.982
RMSEA	0.046
RMSEA 90% CI	0.029–0.060
SRMR	0.276

Note. CFA estimated using WLSMV with ordinal indicators. The high SRMR reflects limited reproduction of the observed correlation matrix, likely due to extreme ceiling effects in some dichotomous items.

## Data Availability

Data will be made available upon request.

## Supplementary Materials

The following supporting information can be downloaded at: https://www.mdpi.com/article/10.3390/healthcare14172673/s1, Table S1. Descriptive characteristics of the sample; Table S2. Descriptives of ELSISF items and Cronbach’s alpha if item deleted; Table S3. Spearman’s correlations among ELSISF total score and subscales, and sociodemographic and health-related variables (education, occupation, quality of life, and physical fitness); Table S4. Mean differences (ANOVA) in ELSISF items between categories of physical and mental health.

## Abbreviations

The following abbreviations are used in this manuscript:

CFA	Confirmatory Factor Analyses
EFA	Exploratory Factor Analyses
ELSI_SF_	Economic Living Standards Index Short Form
IFIS	International Fitness Scale
MCS	Mental Component Summary
PCS	Physical Component Summary
SD	Standard Deviation
WHO	World Health Organization
WLSMV	Weighted Least Squares Mean and Variance-Adjusted
