# Supplementary material for: Preliminary Psychometric Evaluation of the Economic Living Standards Index Short Form (ELSI-SF) Among Spanish Older Adults
Source: Healthcare (Basel). 2026 Aug 22;14(17):2673. doi: 10.3390/healthcare14172673 (PMC13565553; doi:10.3390/healthcare14172673)
Supplement: Supplementary file 1 [file healthcare-14-02673-s001.zip › healthcare-4454441-supplementary.pdf]

SUPPLEMETARY MATERIAL

Table S1. Descriptive characteristics of the sample.

Baseline characteristic	Total *N*= 133
Age (years)	66.2 (5.6)
Sex	
Man	61 (46.2)
Woman	71 (53.8)
Marital status	
Married	74 (69.2)
Unmarried	8 (7.5)
Divorced	9 (8.4)
Widow	10 (9.3)
Unmarried partner	5 (4.7)
Occupation	
Unemployed	20 (15.5)
Employed	27 (22.3)
Entrepreneur/self-employed	9 (7.4)
Pre-retired	8 (6.6)
Retired	54 (44.6)
Study level	
No studies	1 (0.8)
Primary studies	60 (46.2)
Secondary studies	27 (20.8)
Professional training or pre-university studies	26 (20)
University studies	13 (10.5)
Master's or doctoral studies	2 (1.5)
$ELSI_{SF}$ score	23.0 (3.9)
$ELSI_{SF}$ categories[1]	
1. Severe hardship (0 – 8)	0 (0)
2. Significant hardship (9 – 12)	1 (0.8)
3. Some hardship (13 – 16)	10 (7.9)
4. Fairly comfortable (17 – 20)	18 (14.3)
5. Comfortable (21 – 24)	44 (34.9)
6. Good (25 – 28)	51 (40.5)
7. Very good (29 – 31)	2 (1.6)
General fitness[2]	3.38 (0.75)
Health-related quality of life[3]	
Physical health	47.1 (9.2)
Mental health	53.2 (9.0)

Note. Data are presented as *n* and % except for age, $ELSI_{SF}$ score, general fitness, and quality of life, presented in *M* and *SD*.

[1]Categories in Jenssen, et al. (2005).

[2]General physical fitness measured by International Fitness Scale (IFIS), range 1-5.

[3]Health related quality of life measured by the SF-12 questionnaire (Ware et al., 1996)

Table S2. Descriptives of ELSI$_{SF}$ items and Cronbach's alpha if item deleted.

Item	M (SD)	Cronbach's Alpha if item deleted
1. Telephone	0.98 (0.15)	(0.778)
2. Washing machine	0.99 (0.12)	(0.777)
3. Heating available in all main rooms	0.99 (0.12)	(0.779)
4. A good pair of shoes	0.98 (0.15)	(0.779)
5. A best outfit for special occasions	0.99 (0.12)	(0.778)
6. Personal computer	0.95 (0.21)	(0.776)
7. Home contents insurance	0.97 (0.17)	(0.775)
8. Give presents to family or friends on birthdays, Christmas or other special occasions	0.98 (0.15)	(0.775)
9. Visit the hairdresser once every three months	0.99 (0.09)	(0.779)
10. Have holidays away from home every year	0.94 (0.24)	(0.770)
11. Enough room for family to stay the night	0.99 (0.12)	(0.778)
12. Have a holiday overseas at least every three years	0.74 (0.44)	(0.751)
13. Have a night out at least once a fortnight	0.94 (0.24)	(0.771)
14. Have family or friends over for a meal at least once a month	0.94 (0.24)	(0.771)
15. Gone without fresh fruit and vegetables to help keep down costs	1.84 (0.39)	(0.769)
16. Continued wearing clothing that was worn out because you couldn't afford a replacement	1.74 (0.44)	(0.751)
17. Put off buying clothes for as long as possible to help keep down costs	1.42 (0.62)	(0.758)
18. Stayed in bed longer to save on heating costs	1.91 (0.29)	(0.771)
19. Postponed or put off visits to the doctor to help keep down costs	1.62 (0.54)	(0.771)
20. Not picked up a prescription to help keep down costs	1.81 (0.41)	(0.784)
21. Spent less time on hobbies than you would like to help keep down costs	1.23 (0.61)	(0.758)
22. Done without or cut back on trips to the shops or other local places to help keep down costs	1.39 (0.56)	(0.754)
23. Generally, how would you rate your material standard of living?	2.09 (0.61)	(0.778)
24. Generally, how satisfied are you with your current material standard of living?	2.39 (0.78)	(0.798)
25. How well does your total income meet your everyday needs, accommodation, food, clothing, and other necessities?	2.04 (0.80)	(0.762)

M: Mean; SD: Standard deviation

Table S3. Spearman's correlations among ELSI$_{SF}$ total score and subscales, and sociodemographic and health-related variables (education, occupation, quality of life, and physical fitness).

Variable	1	2	3	4	5	6	7	8	9
1. ELSI$_{SF}$ Total	-								
2. ELSI$_{SF}$ Ownership restrictions	.40**	-							
3. ELSI$_{SF}$ Social participation restrictions	.65**	.50**	-						
4. ELSI$_{SF}$ Economising	.86**	.31**	.54**	-					
5. ELSI$_{SF}$ Self-ratings	.70**	.30**	.43**	.33**	-				
6. Studies	.35**	.27**	.31**	.28**	.26**	-			
7. Occupation[1]	.16	-.08	.13	.14	.14	.04	-		
8. Physical health[2]	.19**	.20*	.27**	.17*	.16	.09	.11	-	
9. Mental health[2]	.21**	.02	.26*	.21**	.19*	.05	.15	.11	-
10. General fitness[3]	.25**	.26**	.31**	.27**	.17	.11	.15	.43**	.34**

Note. Data are presented as Spearman coefficients. 1. Assessed with Spanish Society of Epidemiology scale; 2. Assessed with the 12-Item Short Form Health Survey; 3. Assessed with the International Fitness Scale.

*$p < .05$. **$p < .01$

Table S4. Mean differences (ANOVA) in $ELSI_{SF}$ items between categories of physical and mental health.

	Physical health					Mental Health				
**Variable**	**Low**	**Middle**	**Hight**	**p**	**η²**	**Low**	**Middle**	**Hight**	**p**	**η²**
$ELSI_{SF}$ total	22.09 (4.41)	22.96 (3.66)	24.02 (3.19)	.062	.044	21.70 (4.18)	23.56 (3.74)	23.89 (3.27)	.013	.065
$ELSI_{SF}$ 1	1.00 (0.00)	0.95 (0.21)	0.98 (0.15)	.375	.015	0.98 (0.15)	1.00 (0.01)	0.95 (0.21)	.366	.015
$ELSI_{SF}$ 2	0.98 (0.15)	0.98 (0.15)	1.00 (0.01)	.611	.008	0.96 (0.21)	1.00 (0.00)	1.00 (0.00)	.149	.029
$ELSI_{SF}$ 3	0.98 (0.15)	0.98 (0.15)	1.00 (0.00)	.495	.008	0.96 (0.21)	1.00 (0.00)	1.00 (0.00)	.149	.029
$ELSI_{SF}$ 4	0.95 (0.21)	0.98 (0.15)	1.00 (0.00)	.362	.016	0.98 (0.15)	1.00 (0.00)	0.95 (0.21)	.366	.015
$ELSI_{SF}$ 5	0.98 (0.15)	1.00 (0.00)	0.98 (0.15)	.601	.008	1.00 (0.00)	0.95 (0.21)	1.00 (0.00)	.121	.032
$ELSI_{SF}$ 6	0.91 (0.29)	0.96 (0.21)	1.00 (0.00)	.123	.032	0.93 (0.25)	0.98 (0.15)	0.95 (0.21)	.640	.007
$ELSI_{SF}$ 7	0.93 (0.04)	1.00 (0.00)	0.98 (0.15)	.162	.028	0.97 (0.21)	0.98 (0.15)	0.98 (0.15)	.809	.003
$ELSI_{SF}$ 8	0.93 (0.25)	1.00 (0.00)	1.00 (0.00)	.045	.047	0.98 (0.15)	0.97 (0.15)	0.98 (0.15)	.999	.000
$ELSI_{SF}$ 9	0.98 (0.15)	1.00 (0.00)	1.00 (0.00)	.367	.015	1.00 (0.00)	1.00 (0.00)	0.98 (0.15)	.360	.015
$ELSI_{SF}$ 10	0.87 (0.32)	0.96 (0.21)	0.98 (0.15)	.176	.026	0.89 (0.34)	0.98 (0.15)	0.98 (0.15)	.046	.046
$ELSI_{SF}$ 11	1.00 (0.00)	0.98 (0.15)	0.98 (0.15)	.611	.008	0.98 (0.15)	1.00 (0.00)	0.98 (0.15)	.621	.007
$ELSI_{SF}$ 12	0.61 (0.49)	0.78 (0.42)	0.84 (0.37)	.041	.048	0.59 (0.50)	0.84 (0.37)	0.82 (0.39)	.009	.069
$ELSI_{SF}$ 13	0.91 (0.29)	0.91 (0.29)	1.00 (0.00)	.124	.032	0.91 (0.28)	0.95 (0.21)	0.95 (0.21)	.645	.007
$ELSI_{SF}$ 14	0.86 (0.35)	0.96 (0.21)	1.00 (0.00)	.023	.057	0.89 (0.31)	0.95 (0.21)	0.98 (0.15)	.211	.024
$ELSI_{SF}$ 15	1.80 (0.46)	1.86 (0.34)	1.86 (0.35)	.637	.007	1.78 (0.42)	1.91 (0.29)	1.84 (0.43)	.298	.018
$ELSI_{SF}$ 16	1.66 (0.48)	1.77 (0.42)	1.80 (0.41)	.296	.019	1.65 (0.48)	1.77 (0.43)	1.82 (0.39)	.160	.028
$ELSI_{SF}$ 17	1.30 (0.67)	1.44 (0.55)	1.52 (0.63)	.216	.023	1.30 (0.63)	1.47 (0.63)	1.50 (0.59)	.278	.020
$ELSI_{SF}$ 18	1.93 (0.25)	1.84 (0.37)	1.95 (0.21)	.162	.028	1.83 (0.38)	1.95 (0.21)	1.95 (0.21)	.050	.045
$ELSI_{SF}$ 19	1.61 (0.54)	1.60 (0.54)	1.66 (0.57)	.869	.002	1.61 (0.54)	1.56 (0.63)	1.71 (0.46)	.477	.012
$ELSI_{SF}$ 20	1.79 (0.41)	1.75 (0.43)	1.88 (0.39)	.288	.019	1.80 (0.40)	1.81 (0.45)	1.81 (0.40)	.977	.000
$ELSI_{SF}$ 21	1.14 (0.67)	1.24 (0.61)	1.30 (0.55)	.462	.012	1.15 (0.60)	1.26 (0.58)	1.27 (0.66)	.600	.008
$ELSI_{SF}$ 21	1.34 (0.57)	1.33 (0.56)	1.50 (0.55)	.291	.019	1.20 (0.54)	1.42 (0.59)	1.57 (0.50)	.006	.076
$ELSI_{SF}$ 23	2.09 (0.64)	2.02 (0.62)	2.16 (0.57)	.583	.008	1.98 (0.61)	2.14 (0.60)	2.16 (0.61)	.312	.018
$ELSI_{SF}$ 24	2.30 (0.79)	2.40 (0.75)	2.48 (0.79)	.548	.009	2.33 (0.76)	2.33 (0.78)	2.52 (0.79)	.391	.014
$ELSI_{SF}$ 25	1.86 (0.90)	2.13 (0.81)	2.11 (0.65)	.212	.024	1.78 (0.84)	2.23 (0.72)	2.11 (0.78)	.021	.058

Note. Data are presented as means and standard error (SE).
$^{*}p < .05$. $^{**}p < .01$.
